# Synergistic effect of PB2 283M and 526R contributes to enhanced virulence of H5N8 influenza viruses in mice

**DOI:** 10.1186/s13567-017-0471-0

**Published:** 2017-10-25

**Authors:** Xiao Wang, Sujuan Chen, Dandan Wang, Xixin Zha, Siwen Zheng, Tao Qin, Wenjun Ma, Daxin Peng, Xiufan Liu

**Affiliations:** 1 College of Veterinary Medicine, Yangzhou University, Yangzhou, Jiangsu 225009 PR China; 2 Jiangsu Co-Innovation Center for the Prevention and Control of Important Animal Infectious Disease and Zoonoses, Yangzhou, Jiangsu China; 3 Jiangsu Research Centre of Engineering and Technology for Prevention and Control of Poultry Disease, Yangzhou, Jiangsu China; 4 Department of Diagnostic Medicine/Pathobiology, Kansas State University, Manhattan, KS USA

## Abstract

Highly pathogenic avian influenza (HPAI) H5N8 virus has caused considerable economic losses to poultry industry and poses a great threat to public health. Our previous study revealed two genetically similar HPAI H5N8 viruses displaying completely different virulence in mice. However, the molecular basis for viral pathogenicity to mammals remains unknown. Herein, we generated a series of reassortants between the two viruses and evaluated their virulence in mice. We demonstrated that 283M in PB2 is a new mammalian virulence marker for H5 viruses and that synergistic effect of amino acid residues 283M and 526R in PB2 is responsible for high virulence of the HPAI H5N8 virus. Analysis of available PB2 sequences showed that PB2 283M is highly conserved among influenza A viruses, while PB2 526R presents in most of human H3N2 and H5N1 isolates. Further study confirmed that the residues 283M and 526R had similar impacts on an HPAI H5N1 virus, suggesting that influenza viruses with both residues may replicate well in mammalian hosts. Together, these results present new insights for synergistic effect of 283M and 526R in PB2 of H5 HPAI virus on virulence to mammalian host, furthering our understanding of the pathogenesis of influenza A virus.

## Introduction

Since H5N1 highly pathogenic avian influenza (HPAI) virus was first detected from sick goose in Guangdong province in China in 1996, the HPAI H5N1 virus has caused huge economic loss for poultry industry worldwide, and also infected more than 850 humans since 2003 with approximately 50% fatality rate [1], which has been considered as one of candidates to cause the next human pandemic [2, 3]. In late 2013 and early 2014, an HPAI H5N8 virus belonging to the clade 2.3.4.4 of the A/goose/Guangdong/1/1996 lineage caused a large outbreak in domestic poultry in South Korea [4]. Subsequently, the HPAI H5N8 virus was detected in birds in Asian and European countries and outbreaks in domestic poultry caused by this virus have been reported in China, Japan, Germany, the United Kingdom and the United States during 2014–2016 [5–9]. Further spread of the HPAI H5N8 virus along the migratory route of wild birds is possible, and introduction into other countries could occur [10]. Although no human infection case of H5N8 avian influenza virus has been reported, human infections with the related clade 2.3.4.4 H5N6 viruses have been reported in China [11]. Outbreaks of HPAI H5N8 virus have caused significant economic losses to the poultry industry, and more importantly, its potential threat to public health cannot be neglected.

The polymerase complex composed by PB2, PB1 and PA protein plays a major role in virus efficient growth and virulence in mammals. Multiple amino acid substitutions in the polymerase complex that contribute to the virulence and mammalian adaption have been identified. In several subtypes of influenza A viruses (IAVs), the presence of PB2 627K confers high virulence in experimentally infected animal models such as mice, guinea pigs, and ferrets [12–18], and has led to fatal disease in humans [19, 20]. In addition, two mutations (701N/714R) in PB2 of an H7N7 mouse-adapted variant SC35M and an H5N1 virus Kan-1 enhance polymerase activity and virulence in mice and 701N is also one of important determinants for a duck-origin H5N1 virus [21–23]. Recently, H7N9 viruses carrying PB2 526R, only or coupled with 627K or 701N, show enhanced virulence to mice [24]. Several other residues, including 158G [25], 147T, 339T, 588T [26], 588V [27], 591K [28], 598T/I [29] of PB2 and 97I [30], 353R [31], 224P coupled with 383D [32] of the PA are also found to play important roles in virulence to mice.

Functionally, the IAV polymerase complex is responsible for viral genome transcription and replication. In transcription, primers are generated by “cap snatching” reaction. The PB2 subunit initially binds to the capped pre-mRNAs from the host cells, then PA subunit cleaves it after 10–13 nucleotides, and PB1 performs actual RNA synthesis [33–35]. In contrast, replication proceeds in a primer-independent manner [36]. Some studies demonstrate that PB2 and PA are involved in “cap snatching”, while PB1 contributes to RNA extension [37–40]. Of course, PB2 and PA are also involved in the replication process [41]. Therefore, the mutations of polymerase genes may lead to the differences of viral replication in the hosts.

Recently, we have characterized two novel HPAI H5N8 viruses from eastern China with high similarity of their genetic background exhibited remarkably different virulence in mice [42]. The HPAI H5N8 virus A/goose/Eastern China/CZ/2013 (CZ) is highly virulent, whereas HPAI virus A/duck/Eastern China/JY/2014 (JY) is low virulent, which therefore provide a suitable system to explore the molecular basis of virulence in mammals. In this study, we attempted to identify gene(s) and further amino acid(s) that associated with virulence difference in mice by generating reassortant and mutant viruses. Then we evaluated replication of recombinant viruses in two different origins of cells and their polymerase activity in mammalian cells. The correlation of polymerase activity with virulence was also analyzed.

## Materials and methods

### Ethics statements

All the animal experiments were performed in accordance with the Regulations for the Administration of Affairs Concerning Experimental Animals approved by the State Council of People’s Republic of China. The Jiangsu Administrative Committee for Laboratory Animals approved all animal studies (Permit Number: SYXKSU-2007-0005) according to the guidelines of Jiangsu Laboratory Animal Welfare and Ethics of Jiangsu Administrative Committee of Laboratory Animals.

### Cells and viruses

Human embryonic kidney (293T) and Madin-Darby canine kidney (MDCK) cells were grown in Dulbecco’s modified Eagle’s medium (DMEM, HyClone, USA) supplemented with 10% fetal bovine serum (FBS, Gibical, USA) plus antibiotics. Chicken embryonated fibroblast (CEF) cells were grown in M199 medium (HyClone, USA) with 4% FBS. The cells were incubated at 37 °C with 5% CO_2_.

Two HPAI H5N8 viruses, A/goose/Eastern China/CZ/2013 (CZ) and A/duck/Eastern China/JY/2014 (JY) [42], and one HPAI H5N1 virus, A/mallard/Huadong/S/2005 (S) [43] were used in this study and propagated in specific-pathogen-free (SPF) chicken embryonated eggs. The 50% egg infectious dose (EID_50_) was determined as described previously [44]. All experiments involving live viruses and animals were executed in the biosafety level 3 laboratory and animal facility at Yangzhou University.

### Construction of plasmids

Construction of plasmids for establishing reverse genetics for each virus was performed as described previously [45]. Briefly, eight gene segments (PB2, PB1, PA, HA, NP, NA, M, NS) of CZ or JY were amplified by reverse transcription PCR (RT-PCR) and cloned into the vector pHW2000. Mutations were introduced into the PB2 gene by site-directed mutagenesis using the fast mutagenesis system (TansGen, China). The resulting each plasmid was confirmed by sequencing in order to verify the presence of the introduced mutations and the absence of additional unwanted mutations.

### Virus rescue

A mixture of 293T and MDCK cells was co-transfected with eight plasmids encoding eight genes of each influenza virus using PolyJet transfection reagent (SignaGen, USA) as recommended by the manufacturer. After 72 h, the supernatant was harvested and inoculated into 10-day-old SPF embryonated chicken eggs for propagation of the rescued virus. To ascertain each rescued virus, RNA was extracted from the amplified each virus and each gene segment was amplified by RT-PCR and subjected to sequence to ensure the absence of unwanted mutations and the presence of designed mutations.

### Growth curve

Confluent CEF and MDCK cells were inoculated with selected viruses at a multiplicity of infection (MOI) of 0.01 and incubated at 37 °C with 5% CO_2_ for 1 h. Then cells were washed twice with phosphate-buffered saline (PBS) to remove unbound virus particles and the DMEM medium containing 1% FBS was added. Aliquots of supernatants were collected at 12, 24, 36, 48, 60 and 72 hours post-infection (hpi), and virus titers were determined in CEF cells by calculating the 50% tissue culture infectious doses (TCID_50_) per mL using the method of Reed and Muench [44].

### Luciferase assay

Three polymerase genes (PB2, PB1 and PA) and NP gene were cloned into the vector pCDNA3.1(+) using pEASY-Uni Seamless Cloning and Assembly Kit (TransGene, China). The Kozak sequence (ACCACC) was added into front of ORF of each gene to improve gene expression. 293T cells were transfected with 200 ng of each pcDNA3.1 plasmids expressing PB2, PB1, PA and NP, and 200 ng of the luciferase reporter plasmid p-Luci as well as 20 ng of Renilla plasmid as an internal control using PolyJet transfection reagent. After 24 hpi, cell lysates were prepared and tested using the dual-luciferase reporter assay system (Promega, USA) based on manufacturer’s instructions. Luminescence was assayed using a Synergy 2 Multi-Mode Reader (BioTek, USA), and the relative luciferase value was quantified and normalized it to the Renilla luciferase internal control. Each co-transfection experiment was performed in triplicate.

### Mouse experiment

To determine 50% mouse lethal dose (MLD_50_), groups of five 6-week-old female BALB/c mice (Yangzhou Experimental Animal Center, Yangzhou, China) were lightly anesthetized with Zoletil 50 (10–25 mg/kg) and intranasally infected with serial dilutions of the virus. Clinical signs and mortality were monitored daily and until 14 days. Mice were humanely euthanized and considered as dead if weight loss approached 25% of their initial weight. Viruses with an MLD_50_ > 10^6.5^ were considered to be of low virulence, while viruses with an MLD_50_ < 10^3.0^ were considered to be of high virulence [46]. To evaluate virus replication and virulence, groups of four mice were intranasally infected with 10^6.0^ EID_50_ of each indicated virus. On 3 and 5 days post-infection (dpi), two mice from each group were euthanized, and the tissues including lungs, heart, liver, spleen, kidneys, and brain were collected for virus titration. Each tissue sample (whole organ) was homogenized in 1 mL of PBS containing antibiotics and centrifuged at 6800 rpm for 10 min, and 0.1 mL of a 10-fold serial of diluted supernatant was used to inoculate SPF chicken embryonated eggs. Virus titers were calculated as described previously [44].

### Statistical analysis

Statistical analysis was carried out using IBM SPSS Statistic 20.0 (IBM, USA). Viral loads are expressed as the mean ± standard deviation (SD). Polymerase activity values are expressed as the mean ± SD of the results of three independent experiments. Comparisons of experimental groups were estimated by one-way ANOVA analysis of variance, corrected by the *Bonferroni* post-test to determine significant differences. If a *P* value was found to be less than 0.05, the result was considered statistically significant.

## Results

### Rescued CZ and JY viruses maintain the properties of their parental wild-type viruses

**Figure 1:**
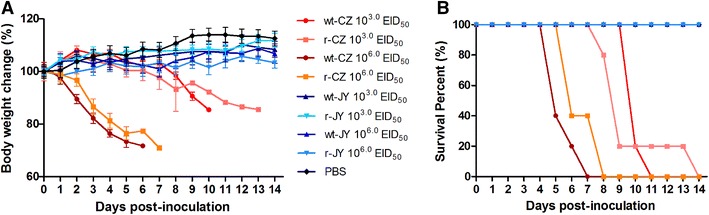
**Virulence of two H5N8 wild-type and their corresponding rescued recombinant viruses in mice.** Six-week-old female BALB/c mice were intranasally inoculated with 10^3.0^ EID_50_ or 10^6.0^ EID_50_ of each indicated virus or 50 μL of PBS as controls. **A** Average body weight of surviving mice in each group (*n* = 5/group) up to 14 dpi are represented as percentages of the original weight on day 0. The error bars represent standard deviations (SD). **B** Survival rate of mice infected with indicated viruses.

**Figure 2:**
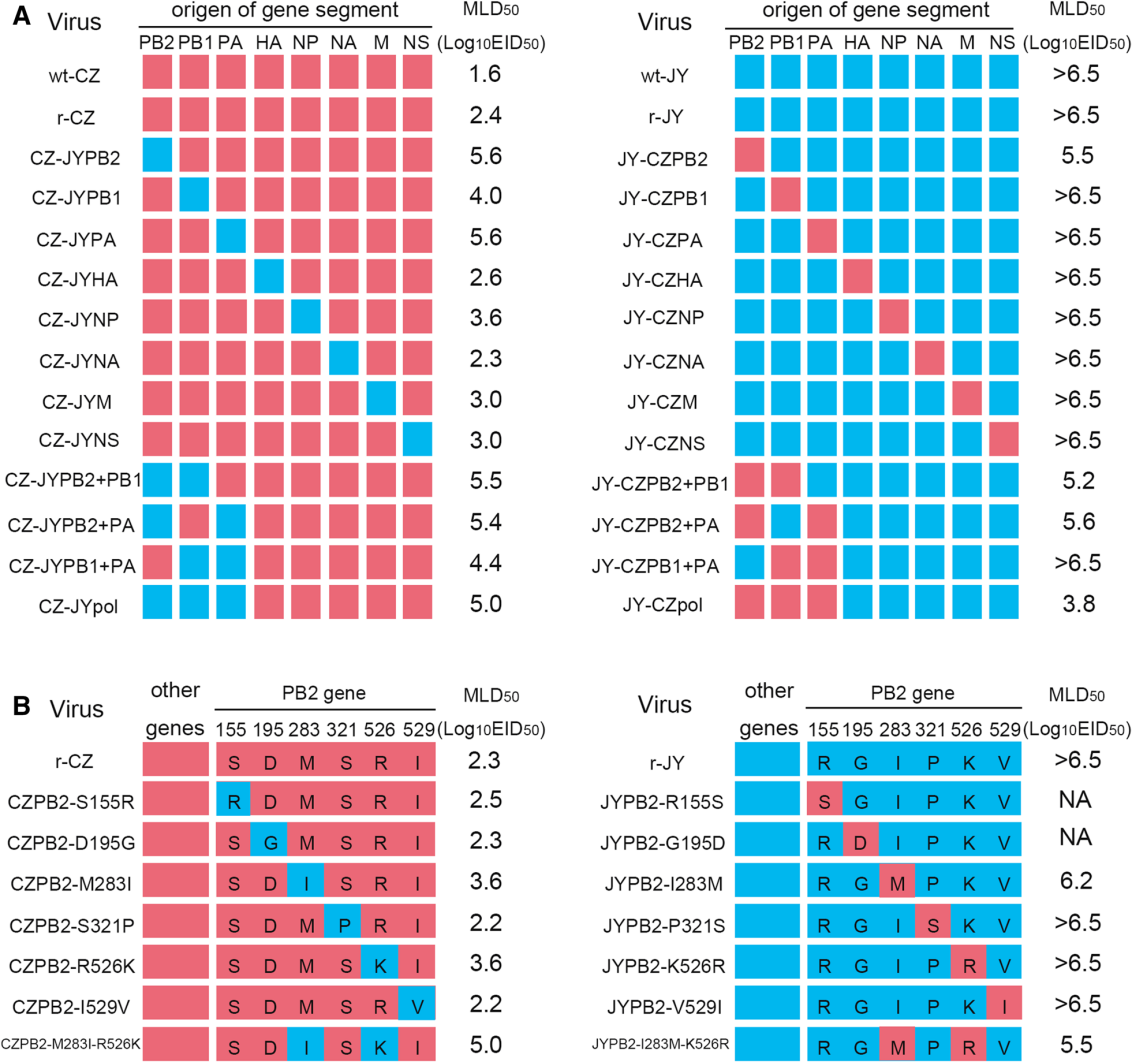
**MLD**_**50**_ **of CZ and JY viruses and their recombinant viruses.** Red and blue boxes indicate the derivation of virus gene segments or PB2 amino acids. MLD_50_ was determined for each indicated virus in 6-week-old female BALB/c mice. **A** MLD_50_ of wild type (wt) or rescued (r) CZ (or JY) virus and its reassortant viruses containing single, double or triple genes from the JY (or CZ) virus. **B** MLD_50_ of r-CZ (or r-JY) and its recombinant viruses with indicated single or double substitutions in the PB2. NA, not available, recused not successful.

Eight-plasmid reverse genetic systems for the CZ and JY viruses were established, and both recombinant viruses (r-CZ and r-JY) were successfully rescued and confirmed by sequence analysis. To determine whether the rescued viruses have the same characteristics as their parental wild-type viruses, a group of mice were intranasally infected with either 10^6.0^ or 10^3.0^ EID_50_ of each rescued virus using their parental viruses as control. Both r-CZ and its parental wt-CZ viruses caused obvious clinical symptoms such as ruffled fur, depression, less activities and obviously severe weight loss and 100% mortality in either dose in mice (Figures 1A and B). In contrast, mice infected with either r-JY or the parental wt-JY virus showed no obvious clinical symptoms, even at a high dose of 10^6.0^ EID_50_ (Figures 1A and B). In addition, MLD_50_ was also determined for both rescued and parental viruses and results showed that the MLD_50_ was 10^2.4^ EID_50_ and 10^1.6^ EID_50_ for the rescued r-CZ and the parental wt-CZ virus, respectively; while the MLD_50_ of both r-JY and wt-JY viruses was above 10^6.5^ EID_50_ (Figure 2A).

Furthermore, we determined viral replication by infecting a group of mice with 10^6.0^ EID_50_ of each rescued and parental virus. Results showed that systemic infections were found in the r-CZ-inoculated mice, evidenced by that virus was detected in heart, liver, spleen, lungs, kidneys and brain on both 3 and/or 5 dpi, which is in consistent with the wt-CZ virus. In contrast, the infections of the r-JY and wt-JY viruses were limited in lungs or occasionally in heart (Table 1). All data demonstrate that the rescued both viruses maintain the properties of their parental wild type viruses in terms of virulence and virus distribution in infected mice.

**Table 1:** **Distribution of CZ and JY viruses and their recombinant viruses in mouse organs at 3 and 5 dpi**

Virus	Virus titer (log_10_EID_50_/1 mL ± SD)											
	Heart		Liver		Spleen		Lung		Kidney		Brain	
	3 dpi	5 dpi	3 dpi	5 dpi	3 dpi	5 dpi	3 dpi	5 dpi	3 dpi	5 dpi	3 dpi	5 dpi
wt-CZ	2.13 ± 0.53	3.63 ± 0.18	1.63 ± 0.18	3.25 ± 0.15	4.00 ± 0.00		5.63 ± 0.53	5.75 ± 0.00	2.25 ± 0.00	3.13 ± 0.18	2.25 ± 0.71	5.38 ± 0.18
r-CZ	1.75 ± 0.00	3.25 ± 0.00	–	3.13 ± 0.53	3.00 ± 0.35	1.50 ± 0.35	5.63 ± 0.18	5.63 ± 0.18	1.38 ± 0.18	3.63 ± 0.18	2.25 ± 0	5.63 ± 0.18
CZ-JYPB2	–	1.75 ± 0.71	–	–	2.00 ± 0.35	1.63 ± 0.18	3.88 ± 0.88	5.25 ± 0.71	–	1.88 ± 0.88	–	1.75 ± 0.71
CZ-JYPB1	1.50 ± 0.35	1.88 ± 0.88	–	–	2.25 ± 0.71	2.00 ± 0.35	4.00 ± 1.06	5.13 ± 0.53	1.75 ± 0.00	1.25 ± 0.00	1.50 ± 0.35	2.75 ± 0.00
CZ-JYPA	1.25 ± 0.00	2.00 ± 0.35	–	–	1.38 ± 0.18	1.50 ± 0.35	5.00 ± 0.35	5.00 ± 0.35	1.38 ± 0.18	1.25 ± 0.00	–	1.38 ± 0.18
CZ-JYHA	1.38 ± 0.18	2.63 ± 0.18	–	–	3.00 ± 0.35	–	4.88 ± 0.53	5.38 ± 0.53	2.13 ± 0.53	1.50 ± 0.35	1.50 ± 0.35	3.50 ± 0.00
CZ-JYNP	1.25 ± 0.00	3.13 ± 0.53	–	–	1.50 ± 0.35	–	4.63 ± 0.53	5.50 ± 0.35	1.38 ± 0.18	2.38 ± 0.18	1.5 ± 0.35	4.38 ± 0.18
CZ-JYNA	2.63 ± 0.18	3.38 ± 0.18	–	1.75 ± 0.71	3.25 ± 0.00	2.13 ± 1.24	5.00 ± 0.35	4.75 ± 0.35	3.25 ± 0.00	2.00 ± 0.35	1.88 ± 0.53	3.50 ± 0.00
CZ-JYM	3.50 ± 0.00	3.25 ± 1.41	–	1.88 ± 0.88	3.50 ± 0.35	2.63 ± 0.18	5.38 ± 0.18	5.75 ± 0.00	1.88 ± 0.53	3.00 ± 0.71	2.63 ± 0.18	4.00 ± 1.06
CZ-JYNS	1.88 ± 0.53	3.00 ± 0.71	–	–	2.25 ± 0.71	–	5.25 ± 0.00	4.13 ± 0.53	2.25 ± 0.00	2.00 ± 0.35	–	3.00 ± 0.71
wt-JY	–	2.93 ± 0.50	–	–	–	–	2.63 ± 0.18	3.63 ± 0.18	–	–	–	–
r-JY	–	–	–	–	–	–	3.75 ± 0.00	3.13 ± 0.88	–	–	–	–
JY-CZPB2	–	3.13 ± 0.53	–	–	2.63 ± 0.18	1.50 ± 0.35	5.50 ± 0.00	4.88 ± 0.53	1.63 ± 0.18	2.00 ± 0.35	–	–
JY-CZPB1	–	–	–	–	–	–	2.88 ± 0.88	3.63 ± 0.18	–	–	–	–
JY-CZPA	–	–	–	–	–	–	3.00 ± 0.71	3.63 ± 0.18	–	–	–	–
JY-CZHA	–	–	–	–	–	–	4.00 ± 0.35	3.00 ± 0.35	–	–	–	–
JY-CZNP	–	–	–	–	–	–	4.50 ± 0.00	3.75 ± 0.35	–	–	–	–
JY-CZNA	–	–	–	–	–	–	3.13 ± 0.53	2.63 ± 0.18	–	–	–	–
JY-CZM	–	–	–	–	–	–	4.38 ± 0.18	3.50 ± 0.00	–	–	–	–
JY-CZNS	–	–	–	–	–	–	4.25 ± 0.71	3.88 ± 0.53	–	–	–	–

### PB2 of the CZ mainly contribute to its high virulence in mice

To identify which gene(s) is (are) responsible for the high virulence in mice, a set of reassortant viruses were generated by exchanging a single gene between r-CZ and r-JY viruses (Figure 2A). MLD_50_ of each reassortant virus was determined in contrast to their parental viruses. When compared to the parental r-CZ virus, CZ-based reassortants carrying a single HA, NP, NA, M or NS gene from the JY virus showed similar MLD_50_ values, whereas the reassortants having PB1, PB2 and PA from the JY virus displayed attenuation (MLD_50_ over 10^4.0^ EID_50_) (Figure 2A). Noticeably, exchange of the single PB2, PB1, PA, HA, NP or NS gene from the JY virus resulted in failure of virus replication of reassortant viruses in liver of infected mice (Table 1). In addition, the virus CZ-JYPB2 could not be detected in mouse heart, kidney and brain tissues at early time point (3 dpi) when compared to the parental r-CZ and other reassortant viruses (Table 1). On the other hand, all JY-based reassortant viruses carrying a single gene segment from the CZ virus, except for PB2 gene, had the same MLD_50_ value as the parental r-JY virus; the reasortant JY-CZPB2 showed an increased virulence in mice and its MLD_50_ was 10^5.5^ EID_50_ (Figure 2A). Interestingly, the JY-CZPB2 could replicate in multiple organs including heart, spleen, lungs and kidneys while the other JY-based reassortant viruses only replicated in mouse lungs (Table 1). These results indicate that polymerase PB2, PB1, or PA gene, especially the PB2 plays a critical role in viral virulence of the HPAI H5N8 virus.

To further clarify whether multiple polymerase genes were required for high virulence of the CZ in mice, reassortant viruses by exchange of double or triple polymerase genes between r-CZ and r-JY viruses were generated and their MLD_50_ were determined (Figure 2A). The CZ-based reassortant viruses with double (CZ-JYPB2 + PB1, CZ-JYPB2 + PA, CZ-JYPB1 + PA) or triple (CZ-JYpol) polymerase genes from the JY virus showed reduced virulence when compared to the parental r-CZ virus. Noticeably, the reassortant CZ-JYPB1 + PA virus showed less attenuation than other 3 reassortant viruses with the PB2 from the JY virus; its MLD_50_ was 10^4.4^ EID_50_ while others were equal to or over 10^5.0^ EID_50_ that is closed to that of the CZ-JYPB2 virus (Figure 2A). On the other hand, the JY-based reassortant viruses with double (JY-CZPB2 + PB1, JY-CZPB2 + PA) or triple (JY-CZpol) polymerase genes from the CZ virus displayed increased virulence when compared to the parental r-JY virus. The reassortant JY-CZPB1 + PA virus showed similar virulence as the r-JY virus with an MLD_50_ over 10^6.5^ EID_50_, while the MLD_50_ of the JY-CZpol was 10^3.8^ EID_50_ (Figure 2A). These results indicate that synergistic effect of polymerase genes plays an important role in virus virulence, but the difference in virulence in mice between CZ and JY viruses is mainly attributable to the effect of the PB2 gene.

### Synergistic effect of PB2 283M and 526R affects viral virulence and tissue tropism

There are six amino acid (S155R, D195G, M283I, S321P, R526K and I529V) differences in the PB2 between CZ and JY viruses. To pinpoint which amino acid(s) is (are) critical for virulence, we generated recombinant viruses with single or double substitutions in the PB2 and tested their virulence in mice (Figure 2B). CZ-based recombinant viruses containing a single substitution at position 283 (CZ-PB2M283I) or 526 (CZ-PB2R526K) of the PB2 showed slightly decreased virulence with an MLD_50_ of 10^3.6^ EID_50_ when compared to the parental r-CZ virus (its MLD_50_ is 10^2.3^ EID_50_), while the CZPB2-M283I-R526K with an MLD_50_ of 10^5.0^ EID_50_ displayed significant attenuation when compared to the parental r-CZ and other recombinant viruses with a single substitution (Figure 2B). Interestingly, single (M283I) or double (M283I and R526K) substitutions in the PB2 resulted in failure of virus replication of the r-CZ virus in mouse tissues including heart, liver, kidney and brain, and only detection in spleen and lungs of infected mice (Table 2). In contrast, the recombinant CZPB2-R526K virus with single R526K substitution was still detected in all organs except for the liver (Table 2). On the other hand, JY-based recombinant viruses containing a single substitution at position 283 of the PB2 (JYPB2-I283M) showed slightly increased virulence with an MLD_50_ of 10^6.2^ EID_50_, while the recombinant viruses including JYPB2-P321S, JYPB2-K526R and JYPB2-V529I showed similar virulence in mice as the parental r-JY virus. Moreover, JYPB2-I283M-K526R displayed enhanced virulence than either JYPB2-I283M or JYPB2-K526R (Figure 2B). Single (P321S, K526R) substitution in the PB2 resulted in change of virus tropism of the r-JY virus, i.e., it could be detected in mouse spleen and brain (JYPB2-P321S), or in the heart (JYPB2-K526R); while single substitutions including I283M and V529I did not change virus tissue tropism which is same as the parental r-JY virus (Table 2). In contrast, the recombinant JYPB2-I283M-K526R virus with double I283M and K526R substitutions was detected in all organs except for the liver and brain (Table 2). All results demonstrate that simultaneous mutations of amino acid at position 283 and 526 in the PB2 have a synergistic effect on viral virulence and tissue tropism.

**Table 2:** **Distribution of CZ and JY viruses and their recombinants with single or double substitutions in the PB2 in mouse organs at 3 and 5 dpi**

Virus	Virus titer (log_10_EID_50_/mL ± SD)											
	Heart		Liver		Spleen		Lung		Kidney		Brain	
	3 dpi	5 dpi	3 dpi	5 dpi	3 dpi	5 dpi	3 dpi	5 dpi	3 dpi	5 dpi	3 dpi	5 dpi
r-CZ	1.88 ± 0.53	2.88 ± 0.53		2.50 ± 0.00	2.63 ± 0.18	1.93 ± 0.13	5.88 ± 0.53	6.40 ± 0.50	1.50 ± 0.35	2.00 ± 0.71	2.00 ± 0.35	5.25 ± 0.00
CZPB2-M283I	–	–	–	–	2.63 ± 0.18	2.00 ± 0.35	4.13 ± 0.58	3.38 ± 0.18	–	–	–	–
CZPB2-R526K	–	2.03 ± 0.03		–	3.13 ± 0.53	2.88 ± 0.53	5.88 ± 0.53	5.88 ± 0.53	1.25 ± 0.00	1.88 ± 0.18	–	2.50 ± 0.35
CZPB2-M283I-R526K	–	–	–	–	1.38 ± 0.53	1.50 ± 0.00	4.63 ± 0.18	2.88 ± 0.88	–	–	–	–
r-JY	–	–	–	–	–	–	3.63 ± 0.13	4.13 ± 0.38	–	–	–	–
JYPB2-I283M	–	–	–	–	–	–	4.63 ± 0.18	4.88 ± 0.53	–	–	–	–
JYPB2-P321S	–	–	–	–	1.25 ± 0.00	–	4.63 ± 0.18	4.13 ± 0.63	–	–	1.38 ± 0.18	–
JYPB2-K526R	–	1.75 ± 0.00	–	–	–	–	4.50 ± 0.00	4.88 ± 0.53	–	–	–	–
JYPB2-V529I	–	–	–	–	–	–	3.38 ± 0.18	3.63 ± 0.18	–	–	–	–
JYPB2-I283M-K526R	–	2.00 ± 0.35	–	–	1.38 ± 0.18	–	6.13 ± 0.53	4.50 ± 0.71	–	1.50 ± 0.35	–	–

### The PB2-I283M-K526R mutation increases replication of the JY in MDCK cells

**Figure 3:**
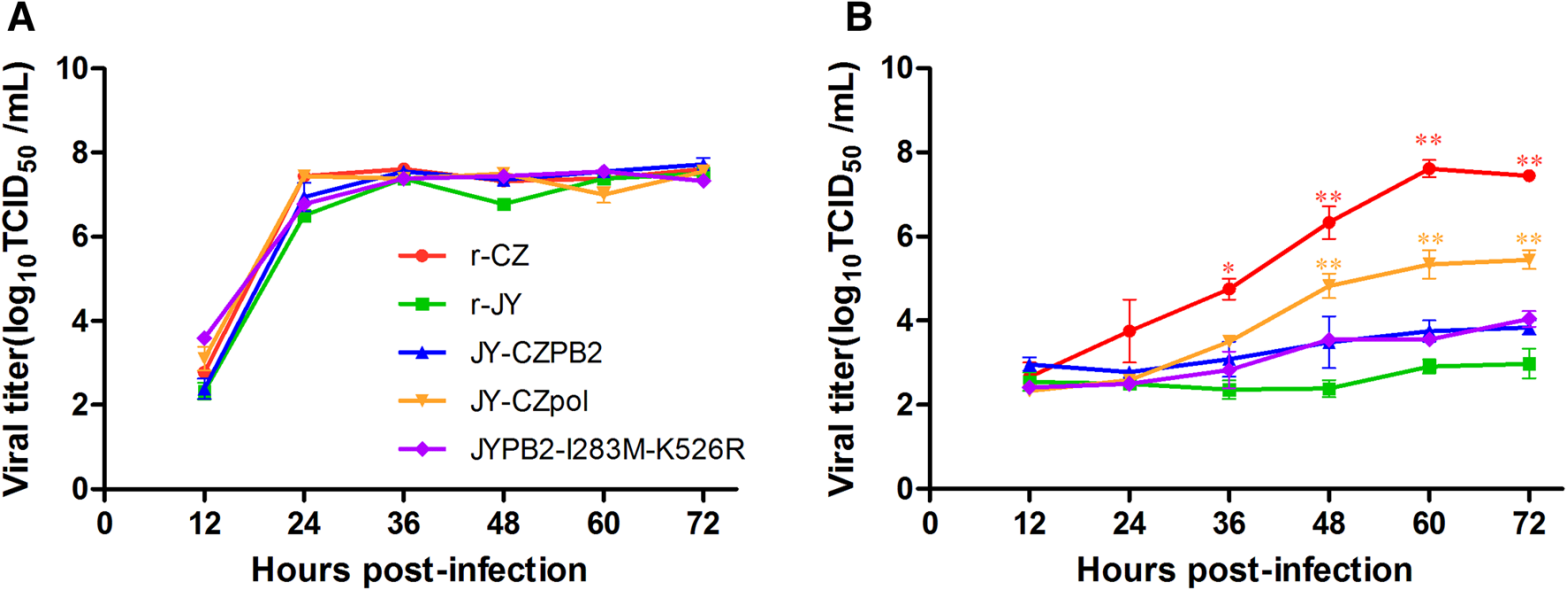
**Growth kinetics of JY virus and its recombinants in CEF and MDCK cells. A** CEF or **B** MDCK cells were inoculated with indicated JY and its recombinant viruses at a multiplicity of infection (MOI) of 0.01 using the r-CZ as a control. Data represent the means of the results from three independent infections (mean ± SD). (*P < 0.05; **P < 0.01 compared to the value of the JY virus).

To determine whether double substitutions I283M and K526R in PB2 influence virus growth dynamics in vitro, we compared growth curves of the r-JY, r-CZ, JY-CZPB2, JY-CZpol and JYPB2-I283M-K526R viruses in both avian-origin CEF and mammalian-origin MDCK cells. All of these viruses grew similarly and efficiently in CEF cells and reached the maximum titers of approximately 10^8.0^ TCID_50_/mL at 36 hpi (Figure 3). In contrast, the r-CZ virus replicated the most efficiently whereas the r-JY virus replicated the least efficiently in all tested viruses in MDCK cells (Figure 3). Both JY-CZPB2 and JYPB2-I283M-K526R viruses replicated in a similar level in MDCK cells but displayed an increased replication than the parental r-JY virus despite of no significant difference observed in virus titers (Figure 3). When introduction of three polymerase genes of the CZ virus into the r-JY virus resulted in enhanced virus replication, significant differences in virus titers were observed between JY-CZpol and the r-JY virus at late time points (48, 60 and 72 hpi) (Figure 3). The results demonstrate that three polymerase genes or single PB2 gene from the CZ virus, or I283M plus K526R substitutions in the PB2 enhance the r-JY virus replication in MDCK cells, not in CEF cells.

### The PB2-I283M-K526R mutation enhances polymerase activity of the JY virus

**Figure 4:**
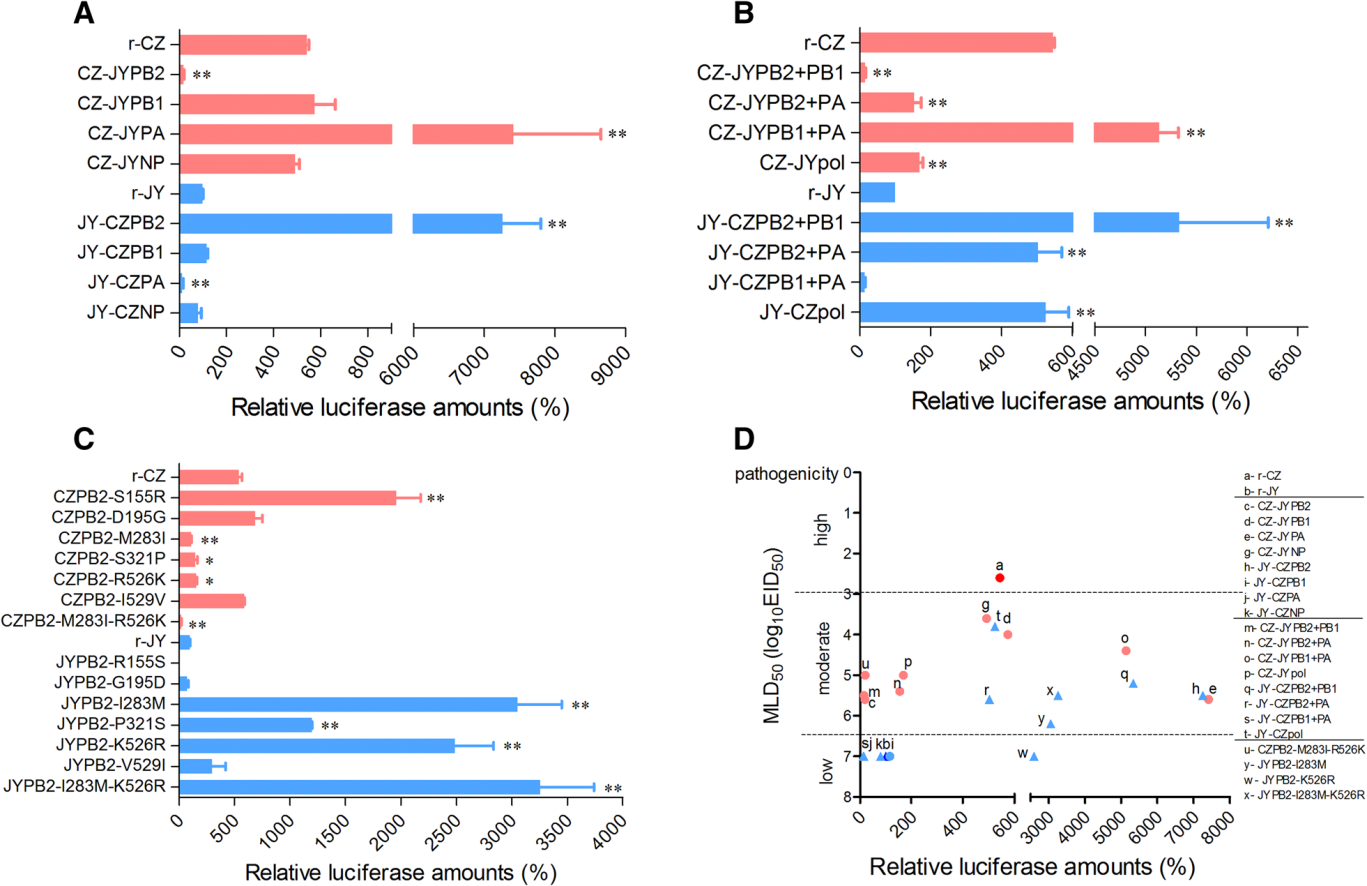
**Polymerase activities of reconstituted RNP complex and correlation of virulence of recombinant virus with polymerase activity.** Comparison of polymerase activities of different ribonucleoprotein complexes with **A** indicated a single polymerase gene or NP from the CZ or JY virus, and with **B** indicated two or three polymerase genes from the CZ or JY virus, and with **C** indicated single or double substitution in the PB2 of the CZ or JY virus. Values represent the mean ± SD of the results of three independent experiments and are standardized to those of the JY (100%). The relative polymerase activity value of each recombinant virus was compared with that of the corresponding parental virus (*P < 0.05; **P < 0.01). **D** Correlation of virulence of recombinant virus with polymerase activity. The MLD_50_ of viruses were plotted (inverted axis) against their polymerase activities.

To understand how three polymerase genes or single PB2 gene from the CZ virus, or I283M plus K526R substitutions in the PB2 affect virus replication, we performed a minigenome assay in 293T cells to investigate polymerase activities of the reconstituted ribonucleoprotein (RNP) complex. As shown in Figure 4A, the relative luciferase amounts of the CZ RNP complex was 6-fold higher than that of the JY RNP complex. When the JY PB2 was introduced into the CZ RNP complex (CZ-JYPB2), the relative luciferase amounts were significantly decreased (29-fold lower). Conversely, the relative luciferase amounts of the JY RNP complex was significantly increased (72-fold higher) by replacing with the CZ PB2 (JY-CZPB2). Interestingly, the PA gene worked in an opposite way in contrast to the PB2 gene. When introduced the JY PA into the CZ RNP complex (CZ-JYPA), the relative luciferase amounts were significantly increased (14-fold higher) when compared to the original CZ RNP complex. Conversely, the relative luciferase amounts of the JY RNP complex were significantly decreased (8-fold lower) by substituting the CZ PA (JY-CZPA). However, replacing either PB1 or NP in both RNP complexes had no effect on the relative polymerase activity (Figure 4A). Replacement of double (JY-CZPB2 + PA, JY-CZPB2 + PB1) or triple (JY-CZpol) polymerase genes from the CZ virus in the JY RNP complex resulted in enhancing the relative polymerase activity, while replacement of double (JY-CZPB1 + PA) polymerase genes from the CZ virus induced a decreased relative polymerase activity in contrast to the original JY RNP complex (Figure 4B). While using the CZ RNP complex as the backbone and replacement of double or triple polymerase genes from the JY virus, the opposite results were obtained (Figure 4B). All results indicate the importance of the CZ PB2 for polymerase activity and virus replication.

We further investigated effects of six different amino acids in the PB2 of both CZ and JY viruses on polymerase activity. Results showed that single substitution (CZPB2-M283I, CZPB2-S321P, CZPB2-R526K) and double substitutions (CZPB2-M283I-R526K) in the CZ PB2 led to significant decrease of relative luciferase amounts in contrast to the unmutated PB2 (Figure 4C). Interestingly, single substitution (CZPB2-S155R) in the PB2 resulted in significant enhancement of relative polymerase activity while another 2 single substitutions (CZPB2-D195G, CZPB2-I529V) in the PB2 did not affect the relative polymerase activity (Figure 4C). Conversely, single substitution (JYPB2-I283M, JYPB2-P321S, JYPB2-K526R) and double substitutions (JYPB2-I283M-K526R) in the JY PB2 resulted in significant increase of relative luciferase activity in contrast to the unmutated PB2 (Figure 4C). Combination of both I283M and K526R in the JY PB2 led to slightly increase of polymerase activity compared to either single substitution. Taken together, PB2-I283M-K526R mutations enhance viral polymerase activity, thereby increasing viral replication in mammalian cells.

### High virulence of the virus requires an optimum polymerase activity

To determine the correlation between virulence and viral polymerase activity, MLD_50_ of the viruses and their corresponding polymerase activities were analyzed and plotted in Figure 4D. When using the JY virus as the genetic backbone, the recombinant viruses with higher polymerase activity showed increased virulence, except for the JYPB2-K526R. The virulent virus was the JY-CZpol whose polymerase activity is relatively lower, same as that of the r-CZ which is the most virulent virus in all tested viruses (Figure 4D). In contrast, the recombinant viruses based on the genetic backbone of the CZ virus, which had a lower or higher polymerase activity, exhibited more reduced virulence when compared to the parental r-CZ virus. Noticeably, the JY-CZPB2 and CZ-JYPA viruses had the equal and highest polymerase activity among all tested viruses, but they only showed a moderate virulence in mice (Figure 4D). Our results indicate that viral virulence is not strictly correlated with polymerase activity, and suggests that there is an optimal level of polymerase activity for high virulence.

### The PB2-I283M-K526R mutation may promote H5 and H3N2 virus adaptation to mammalian hosts

**Figure 5:**
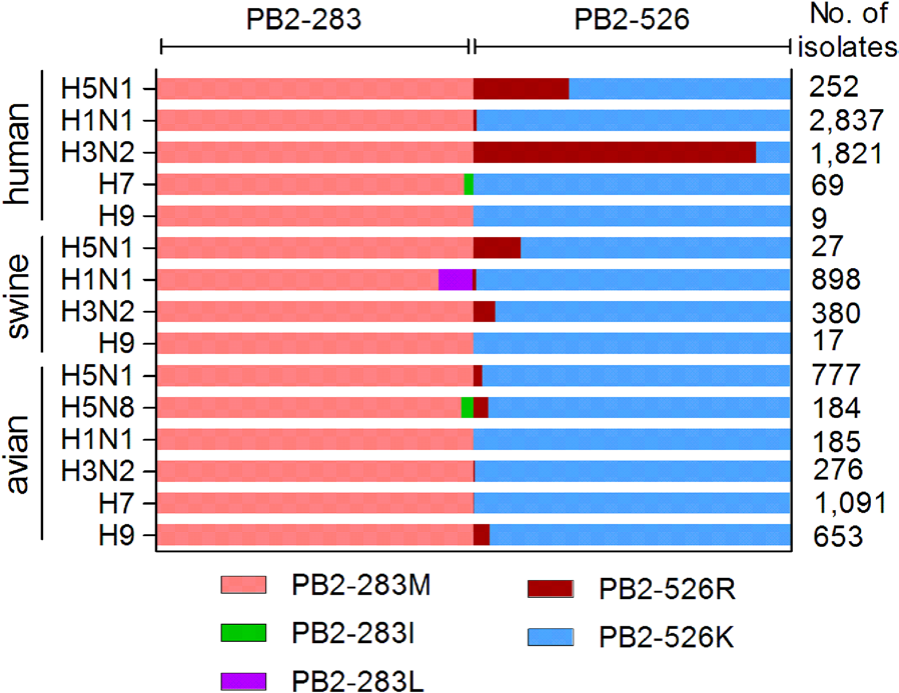
**The frequency of residues at positions 283 and 526 in the PB2 of various subtypes of IAVs isolated from different hosts.** Full-length PB2 sequences of IAVs isolated from avian, swine and human were obtained from the Influenza Sequences Database. Sequence alignment was performed by the Clustal W alignment method using the Megalign program. The percentage of the isolates possessing the indicated residues within each subtype was calculated and indicated by the colored area.

To further investigate the potential role of amino acids at position 283 and 526 in the PB2 in viral adaptation to mammals, we analyzed frequency of these amino acids in the PB2 of various subtypes of IAVs available in the Influenza Sequences Database [47]. Although most of the viruses contain 283M in the PB2, which is highly conserved among IAVs of various subtypes regardless of the host species, some viruses including H5N8 avian isolates, H1N1 swine isolates and H7 human isolates contain either 283I or 238L (Figure 5). In contrast, when PB2 amino acid sequences at position 526 were compared, we could found that most of avian viruses harbor PB2 526K, while PB2 526R are found in some of viruses (Figure 5). The proportion of PB2 526R presented an upward trend from avian to swine and human species in both H3N2 and H5N1 subtype viruses. Especially, the percentage of PB2 526R was up to 89.2% in the human H3N2 viruses, and 30.0% in the human H5N1 isolates (Figure 5). These facts suggest that most likely 526R is an amino acid critical for IAVs’ mammalian adaptation, including humans.

### Synergistic PB2 283M and 526R enhances virulence of an HPAI H5N1 virus in mice

**Figure 6:**
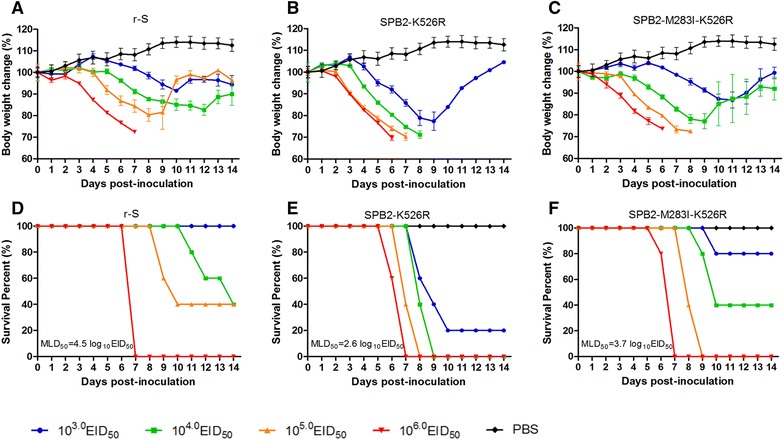
**virulence of HPAI H5N1 S and its mutated viruses in mice.** Six-week-old female BALB/c mice were intranasally inoculated with each indicated virus at a different dose (10^3.0^–10^6.0^ EID_50_) or 50 μL of PBS as controls. Average body weight of surviving mice in each group (*n* = 5/group) up to 14 dpi are represented as percentages of the original weight on day 0. The error bars represent standard deviations (SD). Weight change is depicted for the mice infected with the **A** r-S, the **B** SPB2-K526R and the **C** SPB2-M283I-K526R. Survival rate of mice infected with indicated viruses, the **D** r-S, the **E** SPB2-K526R and the **F** SPB2-M283I-K526R.

To validate whether the two mutations (I283M and K526R) in the PB2 have similar effects on other AIVs, an HPAI H5N1 A/mallard/Huadong/S/2005 (S) virus, which possessed 283M and 526K in the PB2, was selected to generate a recombinant virus with a single substitution (SPB2-K526R) or double substitutions (SPB2-M283I-K526R), and their virulence were evaluated in mice using the parental r-S virus as the control. SPB2-K526R-infected mice developed symptoms such as anorexia, ruffled fur, hunched posture and neurological symptom earlier than the parental r-S-infected or SPB2-M283I-K526R-infected mice. Only the mice infected with the parental r-S virus at high doses of 10^5.0^–10^6.0^ EID_50_ showed severe weight loss (Figure 6A), while the SPB2-K526R even at the doses of 10^3.0^–10^4.0^ EID_50_ also caused obviously severe weight loss in infected mice (Figure 6B). In addition, the SPB2-M283I-K526R at the doses of 10^4.0^–10^6.0^ EID_50_ caused severe weight loss in infected mice (Figure 6C). The SPB2-K526R at the doses of 10^4.0^–10^6.0^ EID_50_ and the SPB2-M283I-K526R at the doses of 10^5.0^–10^6.0^ EID_50_ caused 100% mortality (Figures 6E and F) whereas only a high dose of the parental r-S virus (10^6.0^ EID_50_) led to the death of all infected mice (Figure 6D). These results indicate that the parental r-S virus is moderately virulent for mice (its MLD_50_ is 10^4.5^ EID_50_) and the SPB2-K526R is highly virulent for mice (its MLD_50_ is 10^2.6^ EID_50_). When M283I substitution was introduced into SPB2-K526R, the virulence was reduced (its MLD_50_ is 10^3.7^ EID_50_) compared to SPB2-K526R. This evidence confirmed our discovery that synergistic effect of PB2 283M and 526R enhances virulence of an HPAI H5N1 virus.

## Discussion

Three polymerase genes (PB2, PB1 and PA) of IAV are important for modulating virus polymerase activity, replication kinetics, host specificity and virulence in mammals [22, 32, 48–50]. Several amino acid mutations associated with the virulence or adaptation to mammals of the polymerase proteins have been identified [24, 27, 28, 32, 50–54]. Recently, we have characterized two HPAI H5N8 viruses (CZ and JY) [42] which showed remarkably different virulence in mice despite of only 25 amino acid differences in their whole genome. Through comparing to other highly- and low-virulent avian influenza virus strains, we speculated that the substitutions G195D and I283M in PB2, V339I in PB1, V194I and L422I in HA most likely contribute to the high virulence of the CZ virus in mice [42]. In this study, we have identified the PB2 crucial for difference of virulence in mice between HPAI CZ and JY H5N8 viruses (Figure 2A), and further pinpointed synergistic effect of amino acid residues 283M and 526R in the PB2 responsible for the high virulence of the CZ virus (Figure 2B). This has been demonstrated in the avirulent JY which showed enhanced virulence and extended replication in organs in mice after the CZ PB2 or 283M and 526R double substitutions was introduced. Song et al. [24] reported that an H7N9 virus containing PB2 526R only or coupled with 627K or 701N showed increased virulence in infected mice. Of noticeable is that all the viruses used in their study possess 283M in the PB2 protein. Furthermore, we showed that introduction of the 526R into the PB2 of an HPAI H5N1 S virus (its PB2 has 283M residue) results in enhanced virulence in mice, and when double mutation (M283I plus K526R) were introduced into S virus, its virulence was reduced compared to single mutation (SPB2-K526R) virus. Therefore, synergistic effect of amino acids 283M and 526R in the PB2 plays a critical role in enhancing virulence of influenza virus in mice.

Our studies demonstrate that high virulence in mice and efficient replication in vitro of an influenza virus (such as CZ and JY-CZpol) requires an optimum polymerase activity. In contrast to the recombinant JY-CZPB2 and JYPB2-I283M-K526R viruses in which their vRNPs have much stronger polymerase activity in tested viruses, the polymerase activities of both r-CZ and JY-CZpol vRNPs are much lower but they are much more virulent in mice (Figure 4D) and replicate more efficiently in MDCK cells (Figure 3). Results indicate that influenza virus virulence and replication is not always correlated with the polymerase activity absolutely. This is also corroborated by a prior study in which authors analyzed correlation of polymerase activity of some H7N7 recombinant viruses with virulence and found that three recombinant viruses exhibiting excessive polymerase activity did not show high virulence in mice [22]. In addition, failure to rescue recombinant viruses with a single (155S or 195D) substitution in the PB2 under the background of the JY virus could be due to their low polymerase activities, leading to unsuccess to detect their replication. To date, the mechanisms regarding relationship of viral virulence and polymerase activity remain not completely understood. A reasonable explanation for this phenomenon is that the used mini-genome assay based on luciferase amounts only reflected viral mRNA synthesis but not vRNA levels, as a balanced ratio of viral RNA transcription and replication is critical for virus replication and virulence. Therefore, an assay to measure “real” polymerase activity needs to be developed in order to understand the relationship of viral virulence and polymerase activity.

With the advances in crystallization technologies, the high-resolution structural information of the polymerase protein has been determined [55–57]. PB2 consists of six domains: the N-terminal of PB2 (1–100), the mid domain (248–319), the cap-binding domain, the cap-627 linker (483–538), the 627-domain and the nuclear-localization signal (NLS) domain. The precise cap-binding site is controversial. Honda et al. [58] show that the RNA cap-binding site is located at approximately residues 242–282 and 538–577 of PB2. Another study suggests that the cap binding region is localized to a central region of PB2, with residues 363F and 404F forming the sandwich motif [59]. In addition, positions 318–483 of PB2 as the minimal cap-binding domain was identified [60]. The crystal structure of the polymerase complex shows that the mid and cap-627 linker domains form a rigid unit that is referred to as the mid–link module [61]. The residue 283M is absolutely conserved hydrophobic packing of mid domain and it is one of the crucial residues that maintained the integrity of this module. And the residue 526R is located in the cap-627 linker domain. Therefore, it is reasonable to assume that they can help to stabilize the structure of PB2 or orientation of the cap-binding domain, thereby optimizing viral replication and transcription. Further studies are necessary to verify this assumption.

Based on available influenza PB2 sequences, we show that PB2 283M is highly conserved among various subtypes of IAVs, while 526R is scarce in H3N2 avian influenza viruses, and in H1N1 and H7 viruses isolated from swine, avian or human. However, the percentage of PB2 526R in H3N2 and H5N1 human isolates is 89.2 and 30.0%, respectively. The fact implies that the virus with both 283M and 526R in the PB2 probably has more chance to infect and adapt to humans in nature.

In summary, we have demonstrated synergistic effect of amino acid residues 283M and 526R in the PB2 responsible for enhancing virulence of HPAI H5 viruses in mice. Importantly, residues 283M is highly conserved among various subtypes of IAVs, while residues 526R have been found in the most of human H3N2 viruses that cause human seasonal influenza epidemic, and in many H5N1 human isolates that have been considered to be one of candidates to cause next pandemic [3, 62], suggesting their importance in structure of polymerase and virulence to mammalian. The synergistic effect of 283M and 526R in PB2 may enhance replication of an avian influenza virus in mammalian hosts. Therefore, it warrants attention to give an influenza virus that has the virulence markers during surveillance.
